# Aspirate from human stented saphenous vein grafts induces epicardial coronary vasoconstriction and impairs perfusion and left ventricular function in rat bioassay hearts with pharmacologically induced endothelial dysfunction

**DOI:** 10.14814/phy2.12874

**Published:** 2016-08-01

**Authors:** Helmut R. Lieder, Theodor Baars, Philipp Kahlert, Petra Kleinbongard

**Affiliations:** ^1^ Institut für PathophysiologieUniversitätsklinikum EssenWestdeutsches Herz‐ und GefäßzentrumEssenGermany; ^2^ Klinik für KardiologieUniversitätsklinikum EssenWestdeutsches Herz‐ und GefäßzentrumEssenGermany

**Keywords:** Coronary artery disease, coronary microcirculation, myocardial ischemia, saphenous vein graft, vasoconstriction

## Abstract

Stent implantation into aortocoronary saphenous vein grafts (SVG) releases particulate debris and soluble vasoactive mediators, for example, serotonin. We now analyzed effects of the soluble mediators released into the coronary arterial blood during stent implantation on vasomotion of isolated rat epicardial coronary artery segments and on coronary flow and left ventricular developed pressure in isolated perfused rat hearts. Coronary blood was retrieved during percutaneous SVG intervention using a distal occlusion/aspiration protection device in nine symptomatic patients with stable angina pectoris and a flow‐limiting SVG stenosis. The blood was separated into particulate debris and plasma. Responses to coronary plasma were determined in isolated rat epicardial coronary arteries and in isolated, constant pressure‐perfused rat hearts (±nitric oxide synthase [NOS] inhibition and ±serotonin receptor blockade, respectively). Coronary aspirate plasma taken after stent implantation induced a stronger vasoconstriction of rat epicardial coronary arteries (52 ± 8% of maximal potassium chloride induced vasoconstriction [% KCl_max_ = 100%]) than plasma taken before stent implantation (12 ± 8% of KCl_max_); NOS inhibition augmented this vasoconstrictor response (to 110 ± 15% and 24 ± 9% of KCl_max_). Coronary aspirate plasma taken after stent implantation reduced in isolated perfused rat hearts only under NOS inhibition coronary flow by 17 ± 3% and left ventricular developed pressure by 25 ± 4%. Blockade of serotonin receptors abrogated these effects. Coronary aspirate plasma taken after stent implantation induces vasoconstriction in isolated rat epicardial coronary arteries and reduces coronary flow and left ventricular developed pressure in isolated perfused rat hearts with pharmacologically induced endothelial dysfunction.

## Introduction

Stent implantation into an atherosclerotic lesion of native coronary arteries or aortocoronary saphenous vein grafts (SVG) causes an iatrogenic plaque rupture and results in the release of atherosclerotic particulate debris, microparticles, and soluble vasoactive, thrombogenic, and inflammatory mediators (Kleinbongard et al. 2012, 2013a; Horn et al. 2015). Downstream embolization of such particulate debris obstructs physically the coronary microcirculation and impairs microvascular perfusion and cardiac contractile function (Heusch et al. 2009). The peri‐interventional release of soluble vasoactive mediators contributes to impaired microvascular perfusion (Leineweber et al. 2006; Kleinbongard et al. 2012). The usage of distal occlusion/aspiration protection devices during stent implantation prevents the downstream embolization of particulate debris by withdrawal of the stagnant blood column – the so‐called “aspirate” – including all particulate debris and soluble mediators. Use of distal embolic protection devices is a class I (B) guideline recommendation for percutaneous coronary interventions of SVG (ACC/AHA/SCAI Writing Committee to Update 2001 Guidelines for Percutaneous Coronary Intervention, 2006), but the overall clinical benefit is only modest (Heusch et al. 2009). Management of the acute situation of peri‐interventional microvascular obstruction, however, remains challenging.

Previously we determined soluble vasoconstrictors in the human coronary aspirate plasma and characterized aspirate plasma‐induced vasoconstrictor effects in isolated rat mesenteric artery bioassays (Leineweber et al. 2006; Kleinbongard et al. 2012). Here, we extend our analyses to isolated rat epicardial coronary arteries and the isolated perfused rat heart to analyze the effects of aspirate from human stented SVG on coronary flow and left ventricular developed pressure.

## Materials and Methods

### Study cohort

Samples of nine male patients with symptomatic stable angina pectoris and a stenosis in their SVG were included in this study (Table 1). We used left over samples of the coronary arterial plasma and aspirate plasma from our prior studies (Table 2) (Kleinbongard et al. 2011a,b, 2013a; Baars et al. 2012). The implantation of paclitaxel‐eluting stents attenuates the potential of coronary aspirate plasma to induce vasoconstriction (Kleinbongard et al. 2011b); therefore, only patients treated with bare metal stents were included into this study. All patients were on oral aspirin (100 mg/day) and received 10,000 IU of unfractionated heparin (Ratiopharm^®^, Ulm, Germany) intravenously (for details, see Baars et al. 2012; Kleinbongard et al. 2011a,b, 2012, 2013a). Healthy nonsmoking volunteers without any history of disease or medication (except for oral contraception in women) were recruited (10 men/10 women). The study conforms to the principles of the Declaration of Helsinki and was approved by the Institutional Review Board (No. 14‐5995‐B0). Written informed consent was obtained from all patients and volunteers.

**Table 1:** Patient characteristics (*n* = 9)

Risk factors/comorbidities	
Hypertension	9
Obesity	7
BMI (kg/m^2^)	28.5 ± 1.4
Ex‐smoker	5
Smoker	1
Diabetes mellitus	3
Renal insufficiency	4
Hyperlipidemia	9
Hyperuricemia	4
Laboratory analyses	
Total cholesterol (mg/dL)	159.7 ± 6.9
HDL cholesterol (mg/dL)	47.4 ± 4.2
LDL cholesterol (mg/dL)	87.1 ± 7.2
Triglycerides (mg/dL)	180.9 ± 27.5
Creatinine (mg/dL)	1.2 ± 0.1
Medications	
ACE inhibitors	7
AT1‐receptor antagonists	1
Beta‐blockers	8
Calcium antagonists	4
Statins	6
Diuretics	5
Antidiabetics	5
Aspirin	9
Clopidogrel	5
Prasugrel	2
Saphenous vein graft (SVG)	
Graft age (years)	12.5 ± 2.0
Stenosis diameter (%)	66.0 ± 3.3

Continuous data are presented as mean ± SEM. ACE, angiotensin‐converting enzyme; AT1, angiotensin II type 1; BMI, body mass index; HDL, high‐density lipoprotein; LDL, low‐density lipoprotein.

**Table 2:**
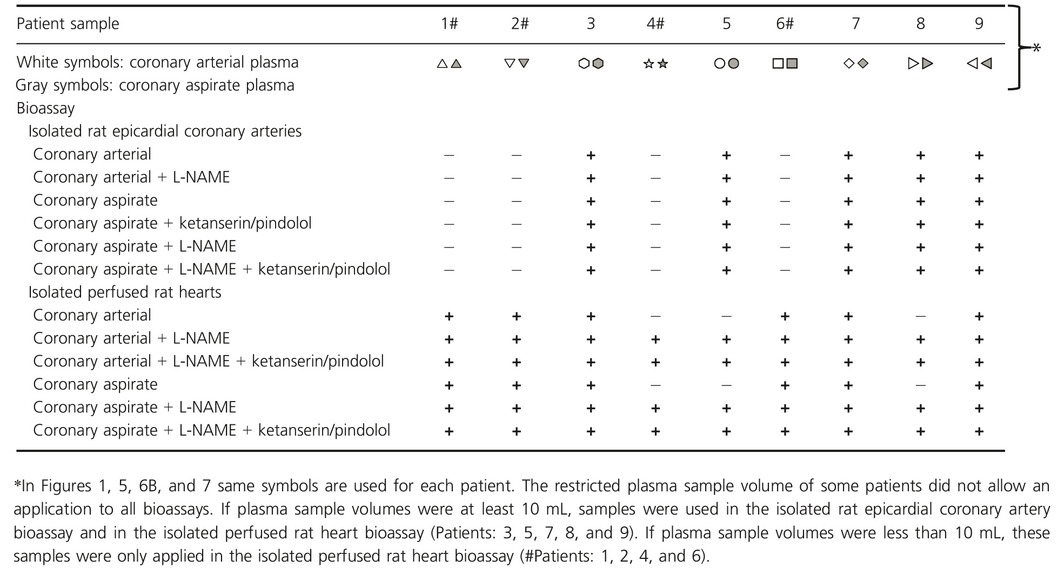
Sample allocation

### Interventional procedure

Saphenous vein grafts stenoses were stented without prior dilatation or debulking to prevent microembolization (Leborgne et al. 2003). A distal balloon occlusion aspiration device (TriAktiv SVG/3.5‐FXTM‐catheter; Kensey Nash, Exton) was used for protection. The balloon was inflated at 2–4 atm with contrast agent before the stent was implanted. After stent implantation the interventional catheter was removed, and the flushing/aspiration catheter was loaded onto the guide wire to aspirate the coronary blood. Subsequently the balloon was deflated. Thrombolysis in myocardial infarction (TIMI) flow was measured before and after stent implantation (The TIMI Study Group, 1989).

### Blood samples, plasma serotonin, and troponin I concentrations

Coronary arterial blood (10 mL into Heparin S‐Monovette, SARSTEDT AG & Co., Nümbrecht, Germany) was taken with the aspiration catheter distal to the lesion before stent implantation and served as control. The coronary aspirate, withdrawn after stent implantation (between 10 and 20 mL), was filtered ex vivo through a 40‐*μ*m mesh filter to separate blood from particulate debris. The coronary aspirate dilution by contrast agent was corrected by reference to the hematocrit. Coronary arterial and coronary aspirate samples were immediately centrifuged (800 × *g*, 10 min, 4°C), and the plasma was quickly frozen in liquid nitrogen and stored at −80°C until further use.

Serotonin in coronary arterial and aspirate plasma was determined using an enzyme immunometric assay kit (Cayman Chemical, Ann Arbor, Michigan) (Kleinbongard et al. 2013a). Troponin I was measured using a specific two‐side immunoassay and detected with the Dimension RxL Max Integrated System (Dimension Flex, Dade Behring GmbH, Marburg and Siemens, Eschborn, Germany) in peripheral venous blood from patients taken before and between 6 and 48 h after stent implantation (Kleinbongard et al. 2011a, 2013a).

Peripheral venous blood samples from healthy volunteers (*n* = 20) were centrifuged without prior filtration, pooled, aliquoted, and stored at −80°C. All plasma samples were thawed at room temperature and centrifuged (15,000 × *g*, 3 min, 4°C) before use.

### Chemicals and drugs

Chemicals and drugs were purchased, if not otherwise declared, from Sigma, Deisenhofen, Germany and were of the purest grade commercially available.

### Animal experiments

All animal experiments were conducted in accordance with the German laws for animal welfare and approved by the Landesamt für Natur, Umwelt und Verbraucherschutz Nordrhein‐Westfalen, Germany (AZ: 8.84‐02.05.20.11.068).

### Isolated rat epicardial coronary arteries

Male Lewis rats (200–350 g, aged 7–11 weeks) were euthanized under enflurane anesthesia. Hearts were excised and 3–6 segments of epicardial left coronary artery segments of 2 mm length were dissected from each heart in ice‐cold oxygenated Krebs–Henseleit buffer (KHB; mmol/l: 119 NaCl, 4.7 KCl, 2.5 CaCl_2_  ×  2 H_2_O, 1.17 MgSO_4_  ×  7 H_2_O, 25 NaHCO_3_, 1.18 KH_2_PO_4_, 0.027 EDTA, 5.5 glucose). The arterial segments were stored in carbogenated (5% CO_2_; 95% O_2_) KHB and equilibrated for 30 min at 37°C. Two steel wires (25 *μ*m in diameter) were inserted into the arterial segments, and the segments were then mounted into an isometric small vessel myograph (Danish Myo Technology, Aarhus, Denmark) (Mulvany and Halpern 1977). The segments were subjected to a wall tension, which was adjusted to the in vivo condition, when the vessel is relaxed and subjected to a transmural pressure of 100 mmHg. This standardized normalization procedure adjusts vascular segments from different vascular territories to a lumen diameter where active force development is maximal (Kleinbongard et al. 2013b). After this normalization procedure, the vascular segments were allowed to stabilize for 30 min and the last 5 min were defined as baseline. The segments were then challenged twice with 0.6 × 10^−1^ mol/L and once with 1.2 × 10^−1^ mol/L potassium chloride (KCl) for 5 min, respectively, and the maximal receptor‐independent vasoconstriction was determined. Endothelial integrity was tested by administration of 10^−4^ mol/L carbachol after preconstriction with 3 × 10^−6^ mol/L serotonin. Isolated coronary artery segments in which carbachol did not reduce serotonin‐induced vasoconstriction by ≥60% were excluded from further experiments. Maximal KCl‐induced vasoconstriction and endothelium‐dependent relaxation were comparable between all groups (Table 3). The diameter of the coronary artery segments was determined during the automated normalization procedure with the Mulvany myograph (Mulvany and Halpern 1977; Kleinbongard et al. 2013b).

**Table 3:**
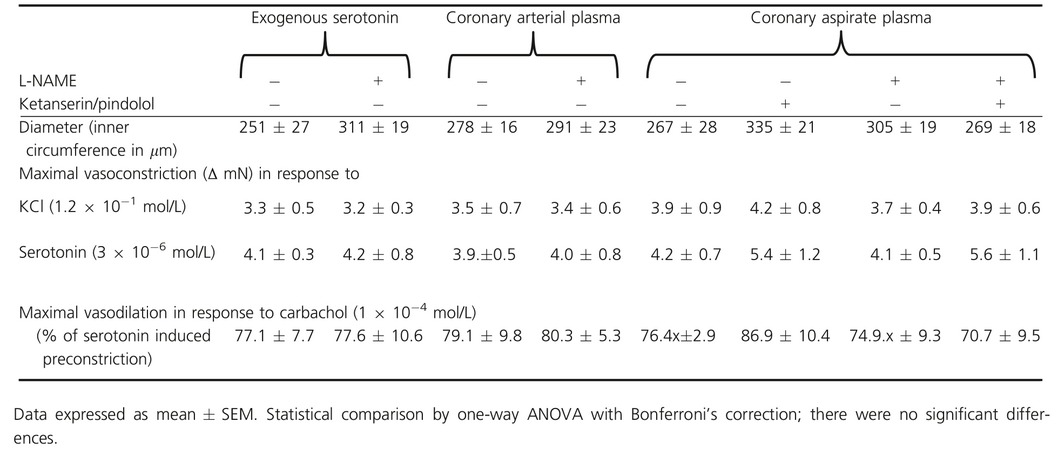
Baseline parameters of isolated rat epicardial coronary arteries

Fifteen minutes prior to the exposure of the vascular segments to coronary arterial plasma or coronary aspirate plasma (dilution of 1:5), the nitric oxide synthase (NOS) inhibitor l‐NG‐nitroarginine methyl ester (L‐NAME, 10^−4^ mol/L) was added to the KHB to simulate endothelial dysfunction in a subset of experiments. Serotonin‐induced vasoconstriction was antagonized by the blockade of serotonin receptors with ketanserin (5‐hydroxytryptamine [5‐HT]_2A/2C_ receptor blocker with *α*_1_‐adrenoceptor blocker effects, 10^−6^ mol/L) and pindolol (*β*‐adrenoreceptor blocker with [5‐HT]_1A/1B_ receptor blocker effects, 10^−7^ mol/L), added to the KHB 15 min prior to coronary arterial or aspirate plasma exposure in a subset of experiments. Usage of this serotonin receptor blockers concentration abrogated the vasoconstrictor response to exogenous serotonin (10^−10^ to 10^−4^ mol/L). The maximal vasoconstrictions of the vascular segments were measured within 8 min after coronary arterial or aspirate plasma exposure as active wall tension (mN) and expressed relative to baseline (ΔmN) and as percent of the maximum KCl‐induced vasoconstriction (% KCl_max_ = 100%). We determined in a subset of experiments cumulative concentration–response curves in response to 1 × 10^−10^ mol/L – 1 × 10^−4^ mol/L for serotonin (±L‐NAME) to scale the aspirate plasma‐induced effects.

### Isolated perfused rat hearts

Male Lewis rats (200–350 g, aged 7–11 weeks) were anesthetized by intraperitoneal pentobarbital (Merial, Hallbergmoos, Germany, 800 mg/kg) supplemented by 1500 IU unfractionated heparin to attenuate coagulation. A bilateral thoracotomy was performed, the heart excised rapidly, mounted on a Langendorff apparatus (Havard Apparatus IH‐SR, Hugo Sachs, March‐Hugstetten, Germany) and perfused at constant pressure of 65–70 mmHg with a modified KHB (in mmol/L: NaCl 118.0, KCl 4.7, MgSO_4_ 1.6, KH_2_PO_4_ 1.2, glucose 5.6, NaHCO_3_ 24.9, sodium pyruvate 2.0, CaCl_2_ 2.0; gassed with 95% O_2_ and 5% CO_2_ in a prewarmed reservoir, ±L‐NAME 10^−4^ mol/L) containing 4.6% bovine albumin (albumin bovine fraction V, SERVA, Heidelberg, Germany). Left ventricular systolic (LVP_sys_) and diastolic pressure (LVP_dia_) were measured with a water‐filled latex balloon inserted into the left ventricle and connected to a pressure transducer (System DPT‐6000, Codan, Lensahn, Germany). LVP_dia_ was adjusted to 5–10 mmHg at baseline by balloon inflation, and heart rate was set to 350 ± 2 bpm by atrial pacing. Coronary flow (CF) was measured with an inline ultrasonic flow probe (Transonic System Inc., New York). The temperature was measured inside the aortic cannula and at the cardiac surface and kept stable at 37°C. The hearts were allowed to stabilize for 20 min. Preparations with CF below 10 mL/min (−L‐NAME) or 8 mL/min (+L‐NAME), respectively, or left ventricular developed pressure (LVDP = LVP_sys_ − LVP_dia_) below 70 mmHg (−L‐NAME) or 60 mmHg (+L‐NAME), respectively, were excluded. Hearts with LVDP above 130 mmHg, CF above 18 ml/min, or arrhythmia duration of more than 3 min were also excluded (Bell et al. 2011). When CF remained stable within a range of ±0.4 mL/min and LVDP within ±5 mmHg, baseline CF and LVDP were recorded for 5 min (Fig. 4A and Table 4). Baseline values used for calculations were averaged over the last 4 min of baseline recording. Plasma aliquots (2 mL) were infused into the perfusate using a syringe pump (Al‐1000, World precision Instruments, Sarasota) at a rate of 2 mL/min. The plasma dilution (ratio of 1:6.4), volume, and timing of its infusion had been elaborated and optimized in preliminary experiments. Perfusion pressure, pH, and oxygen saturation were not affected by plasma infusion.

**Table 4:**
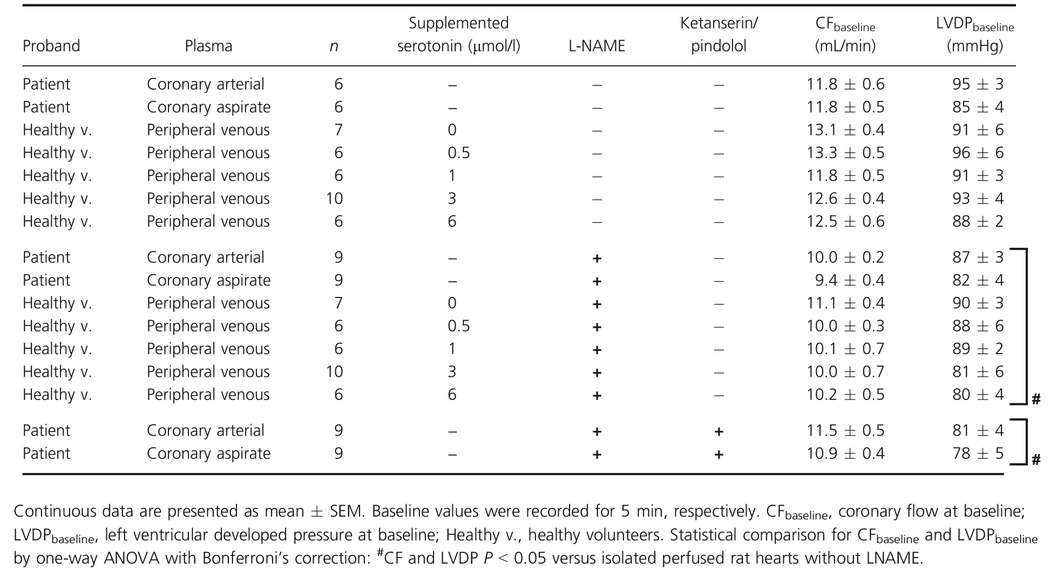
Baseline parameters of isolated perfused rat hearts

Minimal (min) and maximal (max) changes of CF and LVDP were extracted within a time frame of 5 min after plasma infusion and expressed as absolute values after infusion and calculated as Δ mean baseline (Fig. 4A and B). Peripheral venous plasma from healthy volunteers supplemented with increasing concentrations of serotonin (0, 0.5, 1, 3, and 6 μmol/L) was infused into the modified KHB perfusate (±L‐NAME). Plasma supplemented with 0.5 μmol/L serotonin is equivalent to coronary arterial plasma with its mean serotonin concentration. Plasma supplemented with 1.0 μmol/L and 3.0 μmol/L serotonin represents approximately the range of serotonin concentrations measured in the coronary aspirate plasma. To more closely simulate the in vivo serotonin concentration and its effects on CF and LVDP, we corrected the serotonin concentration detected in the coronary aspirate plasma by the application‐dependent dilution in the perfusate and supplemented therefore the plasma from healthy volunteers with a serotonin concentration of 6 μmol/L. Coronary arterial or aspirate plasma, respectively, was infused into the perfusate ±L‐NAME. In a subset of experiments of isolated perfused hearts infused with coronary arterial or aspirate plasma, serotonin receptor antagonists were added to the modified KHB +L‐NAME. The concentrations were equal to those used in the rat epicardial coronary artery segments.

### Statistics

Continuous data are presented as mean ± SEM. Responses of isolated rat epicardial coronary arteries to coronary arterial or aspirate plasma or exogenous serotonin were compared using two‐way repeated measures ANOVA with Bonferroni's correction. Plasma troponin I before and after stent implantation, baseline parameters of isolated perfused rat hearts, serotonin concentrations in coronary arterial and aspirate plasma, changes in CF and LVDP in isolated perfused rat hearts in response to coronary arterial and aspirate plasma, and serotonin‐supplemented peripheral venous plasma from healthy volunteers were compared using one‐way repeated measures ANOVA followed by Bonferroni's correction. Bivariate associations between continuous variables were tested with Pearson's sample correlation coefficients. The statistic tests were performed with Sigma Stat 3.5 (Systat Software Inc., San Jose).

## Results

### Patient characteristics and serotonin release into the coronary aspirate plasma

**Figure 1:**
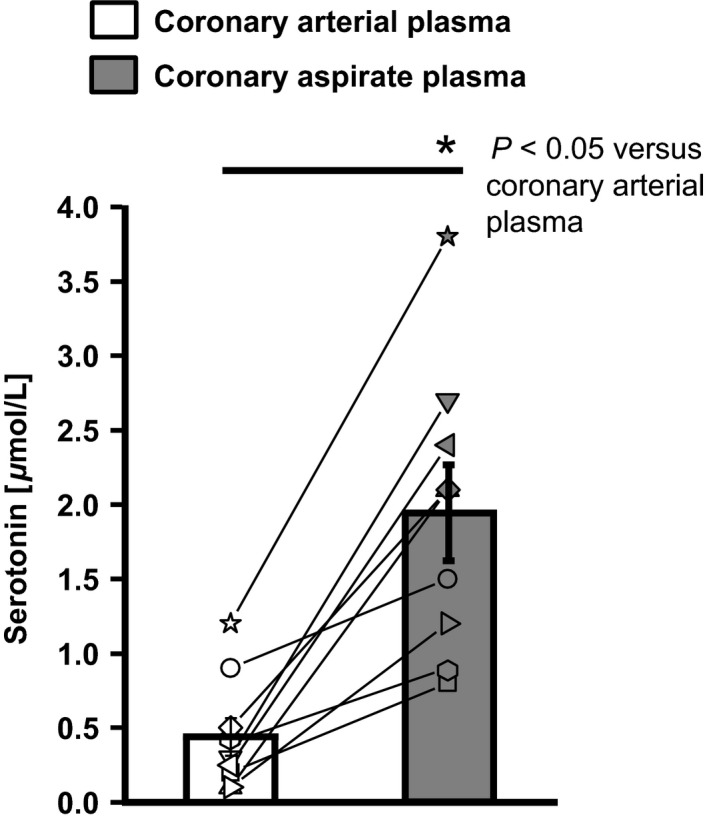
Serotonin concentration in the coronary arterial plasma and in postinterventional coronary aspirate plasma. Statistical comparison by two‐way repeated measures ANOVA with Bonferroni's correction.

Patient characteristics and medications are reported in Table 1. All patients had a history of coronary artery disease and received stent implantation for the first time after the initial coronary artery bypass grafting surgery. TIMI flow was II in 5 and III in 4 patients before the intervention, and it was III in 9 patients after the intervention. Troponin I was 0.03 ± 0.01 ng/mL before and 0.48 ± 0.27 ng/mL (*P *<* *0.05) at its maximum after the intervention. Troponin I exceeded the proposed cut‐off level of 0.15 *μ*g/mL to reflect myonecrosis in two of nine patients (Thygesen et al. 2012). Serotonin concentrations in coronary aspirate plasma increased from 0.4 ± 0.1 μmol/L before to 1.9 ± 0.3 μmol/l (*P *<* *0.05) after stent implantation (Fig. 1).

### Coronary aspirate plasma‐induced vasoconstriction in isolated rat epicardial coronary arteries with and without pharmacologically induced endothelial dysfunction

**Figure 2:**
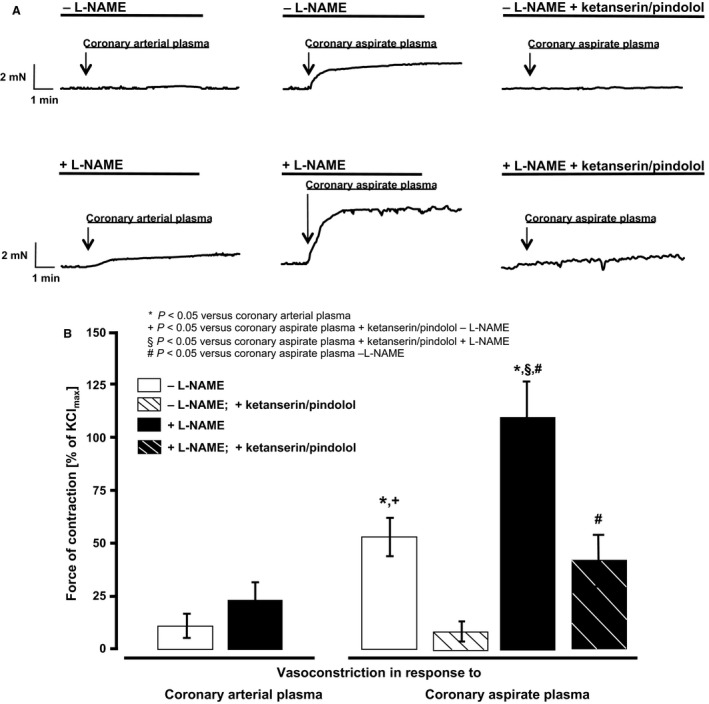
Vasomotor response of isolated rat epicardial coronary arteries to coronary arterial or coronary aspirate plasma. (A) Representative original registrations; (B) vasoconstrictor response of isolated rat epicardial coronary arteries exposed to coronary arterial plasma or coronary aspirate plasma. Statistical comparison by two‐way repeated measures ANOVA with Bonferroni's correction.

Coronary arterial plasma induced a vasoconstriction of 12 ± 8% KCl_max_ and coronary aspirate plasma of 52 ± 8% KCl_max_. NOS inhibition augmented the coronary arterial plasma‐induced vasoconstriction to 24 ± 9% KCl_max_ and coronary aspirate plasma to 110 ± 15% (Fig. 2A and B). Blockade of serotonin receptors attenuated the coronary aspirate plasma‐induced vasoconstriction, independently of NOS inhibition (Fig. 2A and B).

### Concentration–response curves for serotonin in isolated rat epicardial coronary arteries with and without pharmacologically induced endothelial dysfunction

**Figure 3:**
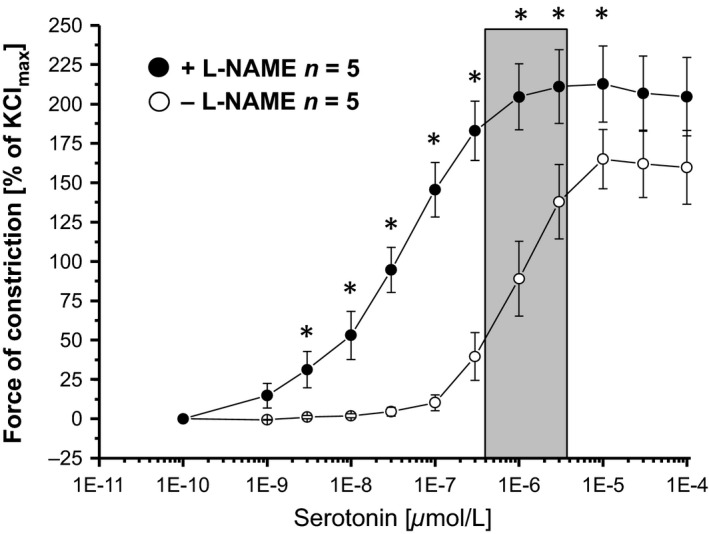
Cumulative concentration–response curves for serotonin in isolated rat epicardial coronary arteries. Vasoconstriction in response to exogenous serotonin, expressed as % of the maximal vasoconstriction induced by KCl. Gray bar: range of the serotonin concentration measured in the coronary arterial and aspirate plasma. *P < 0.0001 versus −L‐NAME. Statistical comparison by two‐way repeated measures ANOVA with Bonferroni's correction.

Exogenous serotonin induced in isolated epicardial rat coronary arteries is a concentration‐dependent vasoconstriction and pharmacological NOS inhibition augmented this vasoconstriction (Fig. 3). Exogenous serotonin in the range of serotonin concentration measured in the coronary aspirate plasma (Fig. 1) induced a roughly comparable vasomotor response compared to that induced by coronary aspirate plasma ±L‐NAME, respectively (cf. response to coronary aspirate plasma in Fig. 2 with the gray box in Fig. 3).

### Coronary aspirate plasma‐induced vasoconstriction and impaired left ventricular function in isolated perfused rat hearts with pharmacologically induced endothelial dysfunction

**Figure 4:**
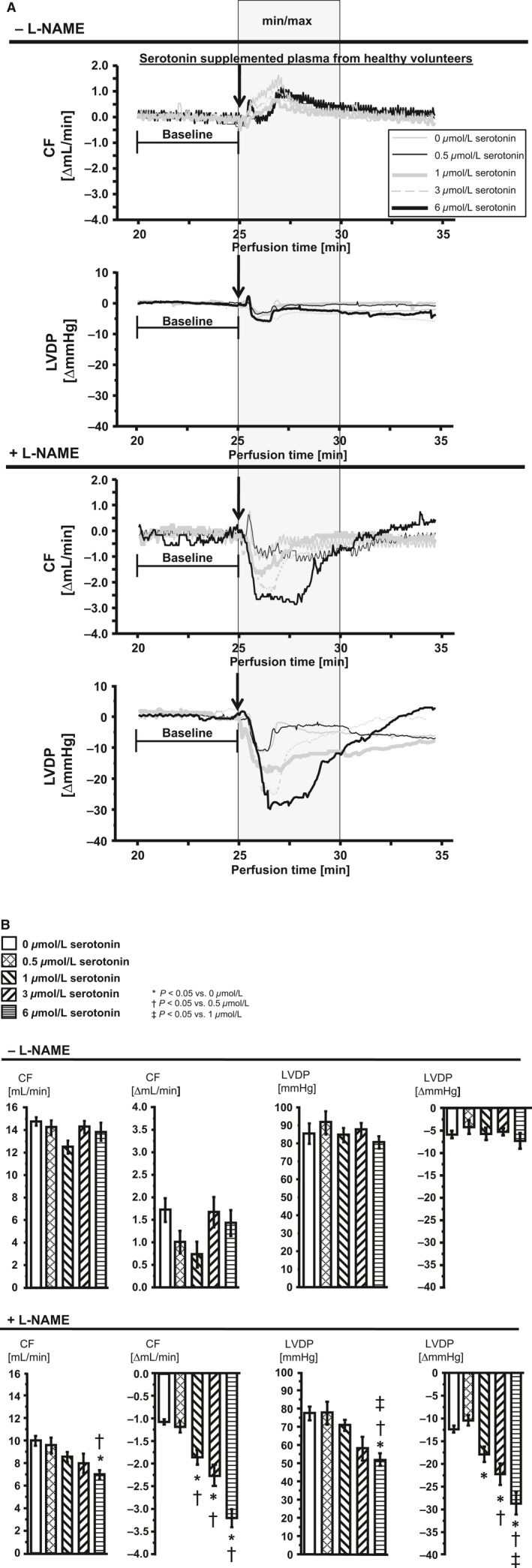
Changes in CF and LVDP in isolated perfused rat hearts after infusion of serotonin‐supplemented peripheral plasma from healthy volunteers. CF, coronary flow; LVDP, left ventricular developed pressure. Supplemented plasma with 0.5 μmol/L is equivalent to coronary arterial plasma with its mean serotonin concentration, 1.0 μmol/L and 3.0 μmol/L represent approximately the range of serotonin concentrations measured in aspirate plasma and 6.0 μmol/L represents the serotonin concentration corrected by dilution in the perfusate. Statistical comparison by one‐way repeated measures ANOVA with Bonferroni's correction. (A) Representative original registrations of changes in CF and LVDP in isolated perfused rat hearts. Plasma infusion (2 mL at a rate of 2 mL/min, plasma diluted by a factor of 6.4) into hearts without and with pharmacological NOS inhibition by L‐NAME. Each original registration approximates the mean min/max value of the group after serotonin‐supplemented plasma infusion. Gray box: time frame in which min and max values were extracted to calculate changes in CF and LVDP, expressed as Δ mean baseline, respectively. (B) Mean of min and max values after plasma infusion are presented as absolute values after infusion and calculated as Δ mean baseline.

The infusion of peripheral venous plasma from healthy volunteers per se induced changes in CF and LVDP (±NOS inhibition) (Fig. 4A and B). CF and LVDP of isolated perfused rat hearts recovered back to baseline after plasma infusion (Fig. 4A and B). Serotonin‐supplemented peripheral venous plasma from healthy controls did not induce serotonin‐dependent changes of CF or LVDP without NOS inhibition, but with NOS inhibition (Fig. 4A and B).

**Figure 5:**
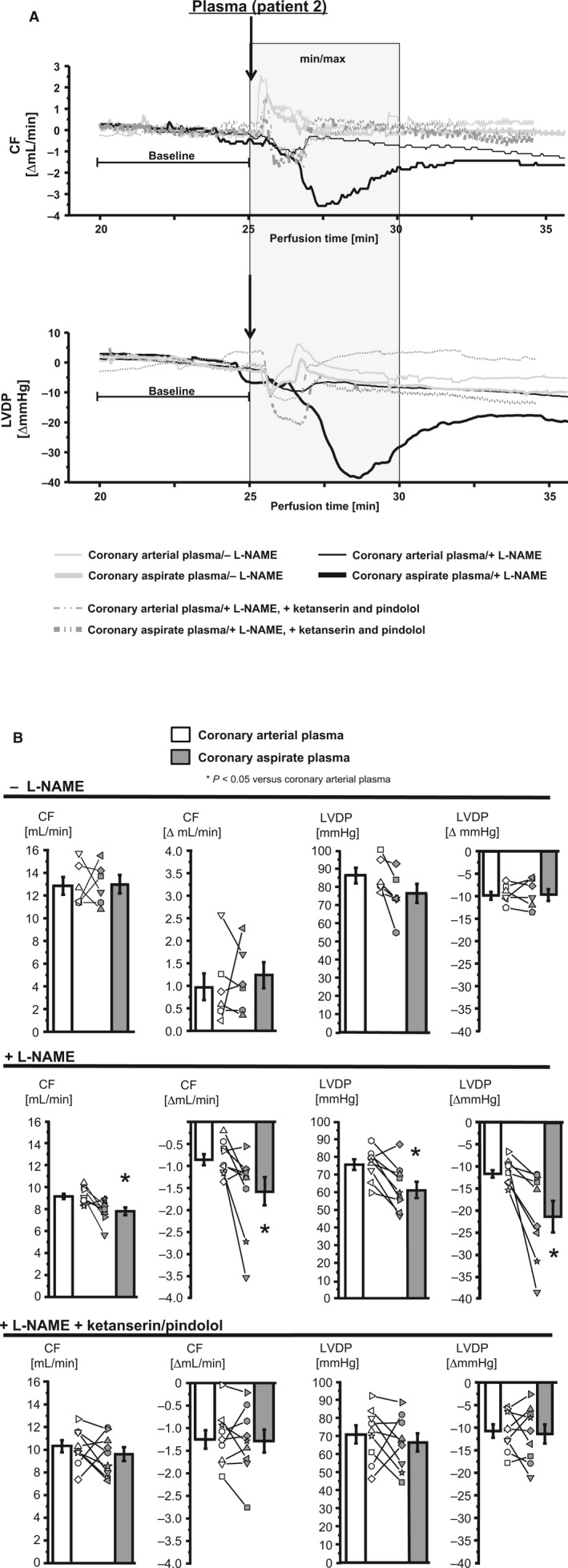
Changes in CF and LVDP in isolated perfused rat hearts after infusion of coronary arterial plasma and coronary aspirate plasma. CF, coronary flow; LVDP, left ventricular developed pressure. (A) Original registrations of changes in CF and LVDP in the isolated perfused rat heart after infusion of coronary arterial plasma and coronary aspirate plasma in Patient 2. Gray box: time frame in which min and max values were extracted to calculate changes in CF and LVDP, expressed as Δ mean baseline, respectively. (B) Mean of min and max values after plasma infusion are presented as absolute values after infusion and calculated as Δ mean baseline. Statistical comparison by one‐way repeated measures ANOVA with Bonferroni's correction.

Changes in CF and LVDP induced by coronary arterial and coronary aspirate plasma were not different without NOS inhibition (Fig. 5A and B). In contrast, with NOS inhibition there was a difference between coronary arterial and coronary aspirate plasma in the reduction of CF and LVDP (Fig. 5A and B). In hearts with NOS inhibition, CF and LVDP reduction induced by the coronary aspirate plasma was stronger than that induced by coronary arterial plasma (coronary aspirate plasma vs. coronary arterial plasma; CF [mL/min]: 7.8 ± 0.4 vs. 9.2 ± 0.2, *P *<* *0.05, LVDP [mmHg]: 61.7 ± 4.2 vs. 75.6 ± 3.0 and CF [∆ mL/min]: −1.6 ± 0.3 vs. −0.9 ± 0.1, *P *<* *0.05, LVDP [∆ mmHg]: −20.2 ± 3.3 vs. −11.2 ± 0.9, *P *<* *0.05) (Fig. 5A and B). The coronary aspirate plasma‐induced effects were transient and disappeared after washout (Fig. 5A). The reduction of CF and LVDP by coronary aspirate plasma was abrogated by the blockade of serotonin receptors (Fig. 5A and B).

**Figure 6:**
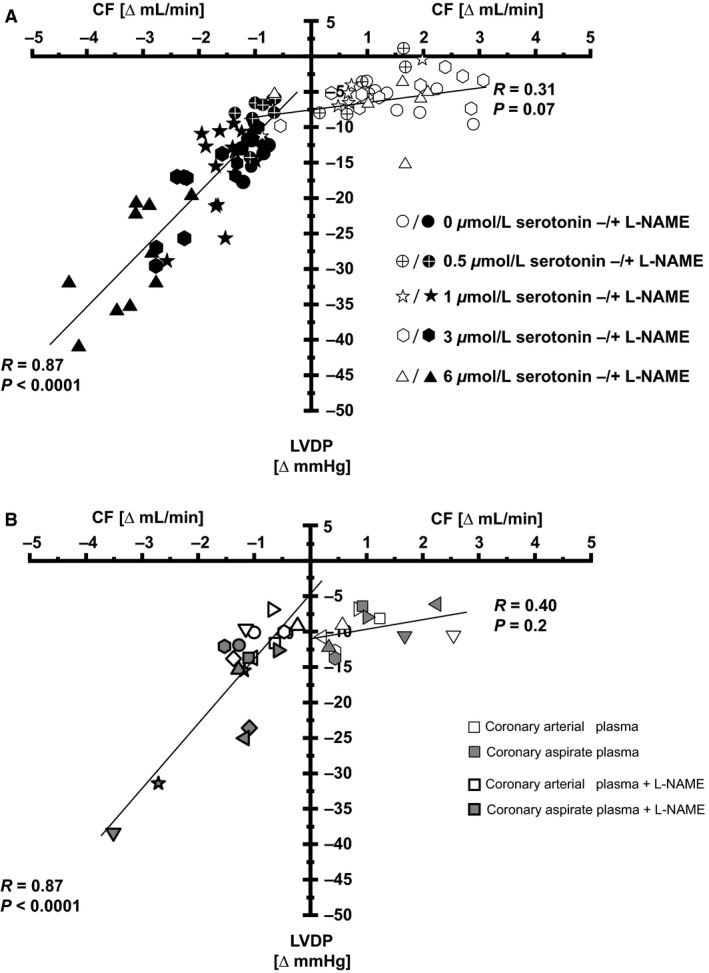
Correlation of CF and LVDP reductions in isolated perfused rat hearts without and with NOS inhibition. CF, coronary flow; LVDP, left ventricular developed pressure. (A) Serotonin‐supplemented peripheral venous plasma from healthy volunteers. (B) Coronary arterial plasma and coronary aspirate plasma without and with NOS inhibition.

Changes in CF correlated with changes in LVDP in response to serotonin‐supplemented peripheral venous plasma from healthy volunteers under NOS inhibition, but not without NOS inhibition (Fig. 6A). Also, changes in CF correlated with changes in LVDP in response to serotonin‐containing (Fig. 1) coronary arterial and coronary aspirate plasma under NOS inhibition, but not without NOS inhibition (Fig. 6B).

**Figure 7:**
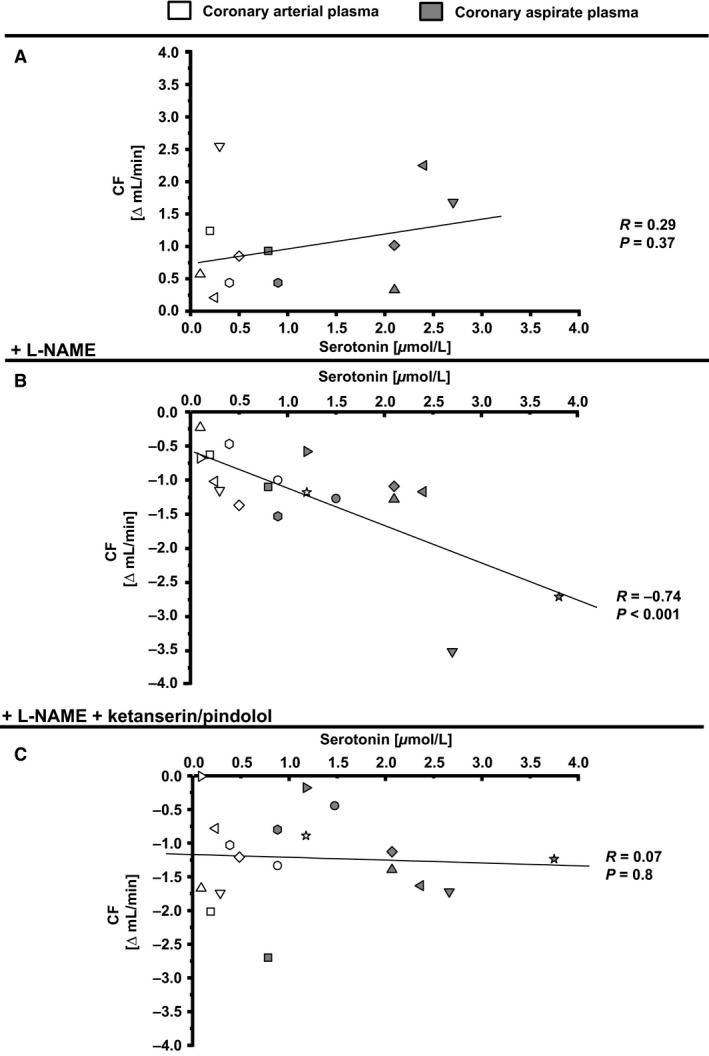
Correlation of serotonin concentration in coronary arterial plasma and coronary aspirate plasma and CF reduction in isolated perfused rat hearts. CF, coronary flow. Values for CF represented as Δ mean baseline.

The measured serotonin concentration in coronary arterial and coronary aspirate plasma (Fig. 1) did not correlate with the changes in CF without NOS inhibition (Fig. 7A). However, with NOS inhibition there was a correlation between the measured serotonin concentration in coronary arterial and aspirate plasma and changes in CF (Fig. 7B). This correlation was abrogated by additional serotonin receptor blockade (Fig. 7C).

## Discussion

### Aspirate plasma induces vasoconstriction and impairs coronary perfusion and left ventricular function in rat heart bioassays with pharmacologically induced endothelial dysfunction

We confirmed that serotonin is released into the coronary circulation during stent implantation into SVG (Kleinbongard et al. 2012) and induces a vasoconstriction in in vitro bioassays. The serotonin release into aspirate plasma of this small patient collective was comparable to that measured in the larger patient collectives of our prior studies (Kleinbongard et al. 2011a, 2013a). We extend our prior data by (1) detecting the vasoconstrictor effects of aspirate plasma in isolated rat epicardial coronary arteries and in the coronary circulation of isolated perfused rat hearts; and (2) revealing functional consequences on left ventricular developed pressure.

The extent of aspirate plasma‐induced vasoconstriction was almost independent of endothelial functionality in our prior studies in isolated rat mesenteric arteries (Kleinbongard et al. 2011a). However, artery segments from different vascular territories differ in their vasomotor properties (Kleinbongard et al. 2013b). We here used isolated rat epicardial coronary arteries instead of mesenteric arteries and indeed the pharmacologic‐induced endothelial dysfunction augmented the aspirate plasma‐induced vasoconstriction. In the isolated perfused rat heart bioassay, however, the reduction of CF in response to aspirate plasma was exclusively detectable under pharmacologically induced endothelial dysfunction and the same was true for reduction of LVDP. This vasoconstriction in isolated epicardial coronary arteries and the CF and LVDP reduction in the isolated perfused heart was abrogated by serotonin receptor blockade with ketanserin ([5‐HT]_2A/2C_ receptor blocker with *α*_1_‐adrenoceptor blocker effects) (Marwood 1994) and pindolol (*β*‐adrenoceptor blocker with [5‐HT]_1A/1B_ receptor blocker effects) (Langlois et al. 1993). Despite the questionable selectivity of ketanserin and pindolol for 5‐HT potentially adrenoceptor‐mediated effects by aspirate plasma seem to be unlikely. In our prior studies, we did not detect a release of epinephrine and norepinephrine into coronary aspirate plasma during stent implantation (Kleinbongard et al. 2011a, 2012). Here, the plasma serotonin concentration and CF reduction correlated in the isolated perfused rat heart bioassay under NOS inhibition. Beside serotonin, endothelin, thromboxane, and TNF*α* are released into the aspirate plasma (Kleinbongard et al. 2011a, 2013a). Endothelin was almost exclusively released into aspirate plasma during stent implantation into native coronary arteries (Kleinbongard et al. 2013a). Released thromboxane and TNF*α*, however, contribute less to aspirate plasma‐induced vasoconstriction in isolated rat mesenteric arteries (Leineweber et al. 2006; Kleinbongard et al. 2011a). In isolated rat epicardial coronary arteries and in the isolated perfused rat heart, the effect of additional released mediators in the aspirate plasma also seem to be of minor relevance. Taken together, these findings underline the potential role of released serotonin as the major vasoconstrictor in the coronary aspirate plasma.

### Serotonin as the mediator for reduction of CF and LVDP in isolated perfused rat hearts with pharmacologically induced endothelial dysfunction

We mimicked the aspirate plasma‐induced effects in the isolated perfused rat heart by infusing serotonin‐supplemented peripheral venous plasma from healthy volunteers. We (1) used concentrations measured in the aspirate plasma; (2) corrected this serotonin concentration for the dilution in our isolated perfused rat heart model; and (3) induced pharmacologically endothelial dysfunction to estimate more closely the potential effect in the patient vasculature. Under pharmacologically induced endothelial dysfunction, the reduction of CF and LVDP by serotonin‐supplemented plasma was dependent on serotonin concentration; the same was true for coronary arterial and aspirate plasma. Considering that in isolated atriums of rats serotonin has apparently no negative inotropic effect (Laer et al. 1998), the change in left ventricular function seems to be secondary to the flow reduction.

Extraluminal exposure of isolated vessels to serotonin‐containing aspirate plasma resulted in a vasoconstriction, confirming prior reports (Martin 1994). Infusion of serotonin‐containing plasma into the isolated perfused rat heart decreased under pharmacological NOS‐inhibition the CF in a concentration‐dependend manner, confirming prior reports of saline serotonin infusion (Mankad et al. 1991). In the healthy coronary vasculature, serotonin induces heterogeneous effects across various species (Martin 1994). The stimulation of endothelial intraluminal 5‐HT_1_ receptors induces a vasodilation which is mediated by endothelial NO release (Vanhoutte et al. 2009), whereas stimulation of smooth vasculature 5‐HT_2A_ receptors mediates a vasoconstriction. A serotonin‐mediated vasoconstriction of epicardial coronary arteries and concomitant vasodilation in the coronary microcirculation is described in different species (Bove and Dewey 1983; Lamping et al. 1989; Martin 1994). This may be related to a different 5‐HT receptor distribution in dependence of the vessel size (Martin 1994). We here blocked both the 5‐HT_1_ and 5‐HT_2_ receptors simultaneously and could therefore not differentiate between the receptor‐specific effects. In the isolated rat epicardial coronary artery bioassay we dissected conductance arteries. Thus, we did not consider the effect of serotonin‐containing aspirate in the coronary microcirculation. The infusion of serotonin‐containing plasma into the isolated perfused rat heart, however, affected both levels of vascular territories. We therefore could not discriminate between specific effects in the coronary macro‐ and microcirculation.

In dysfunctional endothelium with a reduced NOS activity serotonin mediates the release of endothelium‐derived constriction factors and induces a vasoconstriction (Vanhoutte et al. 2009). This serotonin‐dependent vasoconstriction in the coronary circulation is also present in animal in vivo models with atherosclerosis (Chilian et al. 1990), in ex vivo preparations of isolated atherosclerotic human epicardial coronary arteries (Chester et al. 1990), and in vivo during elective coronary angiography in patients with angiographic‐detected coronary atherosclerosis (Golino et al. 1991; Leosco et al. 1999).

### Limitations

Different limitations in our in vitro bioassays may affect the transferability to the situation in the patient:

1. The infusion of diluted aspirate plasma into the buffer stream exposed under high‐flow conditions the whole coronary vasculature of the isolated perfused rat heart bioassay resulting in an additional dilution of the aspirate plasma and potentially attenuating the aspirate plasma‐induced effects. In the clinical situation of stent implantation into SVG in the patient, however, aspirate is released into a restricted myocardial area under low‐flow conditions.
2. We neglected in our bioassays the influence of released particulate debris during stent implantation into SVG (Heusch et al. 2009; Kleinbongard et al. 2012). Both the released soluble and the particulate components may determine CF and/or stagnation and accumulation of debris and mediators in the downstream circulation.
3. Our study is limited to a very small number of patients. Because of the limited sample volume of the coronary arterial and aspirate plasma we were not able to apply all samples in both bioassays.
4. The patient collective we used is characterized by a fully developed symptomatic atherosclerosis and thus endothelial dysfunction is prevalent in these patients (Ludmer et al. 1986; Davignon and Ganz 2004). However, in our bioassays pharmacologically induced endothelial dysfunction simulated only one aspect of the atherosclerosis and neglected the influence of, for example, vascular inflammatory processes or disorders of lipid metabolism (Dart and Chin‐Dusting 1999; Libby 2012). The coronary vasculature of patients might be, therefore, more sensitive to vasoconstrictors (Chester et al. 1990; Vanhoutte et al. 2009). However, on the other hand, the patients received medications, for example, statins (Davignon and Ganz 2004), which are known to attenuate endothelial dysfunction.

### Clinical implications

Although our patients received dual platelet inhibition with aspirin and clopidogrel or prasugrel, we still detected a significant serotonin release. An impaired response to clopidogrel treatment is shown for type 2 diabetes (Angiolillo et al. 2014), renal insufficiency (Gremmel et al. 2013a), and obesity (Gremmel et al. 2013b). The remaining serotonin release in clopidogrel‐treated patients is interindividually different (Muller et al. 2010) and attributed to platelet activation during stent implantation (Bax et al. 1994; Gawaz et al. 1996). Likewise, the release of particulate debris, microparticles, and soluble mediators differs interindividually and depends on the underlying disease and the type of the interventional‐treated vessel (Baars et al. 2012; Kleinbongard et al. 2013a; Horn et al. 2015). Therefore, certain patients may benefit from a more individualized and aggressive platelet inhibition prior to interventions to prevent the release of potentially vasoactive mediators. The decreased incidence of peri‐interventional impaired microvascular perfusion and myocardial infarction during SVG intervention in the contemporary era may be attributed to a more aggressive platelet inhibition (Lee et al. 2011; Brennan et al. 2015).

## Conflict of Interest

None declared.
